# Canine chondrodystrophic intervertebral disc disease (Hansen type I disc disease)

**DOI:** 10.1186/1471-2474-16-S1-S11

**Published:** 2015-12-01

**Authors:** Clare Rusbridge

**Affiliations:** 1 Fitzpatrick Referrals, Halfway Lane, Eashing, Godalming, Surrey, GU7 2QQ, UK; 2 School of Veterinary Medicine, Faculty of Health & Medical Sciences, Duke of Kent Building, University of Surrey, Guildford, Surrey, GU2 7TE, UK

## 

Intervertebral disc disease (IVDD) is the most common spinal disease in dogs. Chondrodystrophic dogs, which have disproportionably short and curved limbs, are predisposed to Hansen type I IVDD and of these the miniature Dachshund is over represented [1,2], is more likely to be presented at an earlier age [3] and is at greater risk of a severe spinal cord injury [4]. The tendency for IVDD in Dachshunds is inherited [5,6] and a major locus on chromosome 12 harbours genetic variations affecting the development of intervertebral disc calcification [7]. Hansen type I disc degeneration is thought to occur because of loss of notochordal cells which produce proteoglycans which “hold water” in the disc. Chondrodystrophic dogs have a primary deficiency of notochordal cells, a study found that large notochordal cells in the nucleus pulposus of chondrodystrophoid dogs formed 13% of the cell population in young dogs and fell to 0.4% in adults, whereas they were the predominant cell type in the nonchondrodystrophoid dogs at all ages [8]. Thus chondrodystrophoid dogs suffer early degenerative changes in the disc and a concomitant reduction in proteoglycan content, increased collagen, and loss of water content making the discs likely to herniate [8]. Certain lifestyle factors may increase or decrease risk of IVDD and this is currently under investigation. There is a suggestion that exercise (including stair climbing) reduces the incidence of disc calcification [9]. By contrast it has been hypothesized that obesity/lack of postural muscle strength may increase risk. Dachshunds with intervertebral disc extrusion had significantly smaller cross sectional area and greater fat infiltration in the epaxial muscles (muscles which lie dorsal to the horizontal septum of the vertebrae which mobilize and globally stabilize the trunk [10]) compared to dogs presenting with fibrocartilagenous embolism (another acute onset intervertebral disc related spinal disease) [11]. In addition chondrodystrophic dogs may have increased risk of IVVD if they have a more extreme conformation e.g. Dachshunds that have a longer back and shorter limbs are more at risk [12] and breeds with a comparatively heavy head such as the Bassett Hound may be more at risk of cervical disc disease. Breeds with a tendency for kyphoscoliosis such as the French Bulldog may be more at risk of IVDD in the IVDs adjacent to a vertebral malformation [13,14]. This presentation details the pathogeneis, clinical presentation, diagnosis and treatment of IVDD in chondrodystrophic dogs.
